# Cardiovascular Risk Estimation in Colombia Using Artificial Intelligence Techniques

**DOI:** 10.1155/crp/2566839

**Published:** 2025-05-11

**Authors:** Jared Agudelo, Oscar Bedoya, Oscar Muñoz-Velandia, Kevin David Rodriguez Belalcazar, Alvaro Ruiz-Morales

**Affiliations:** ^1^Department of Internal Medicine, Universidad Libre, Cali, Colombia; ^2^Department of Systems Engineering and Computer Science, Universidad del Valle, Cali, Colombia; ^3^Department on Internal Medicine, Pontificia Universidad Javeriana, Hospital Universitario San Ignacio, Bogotá, Colombia; ^4^Department of Clinical Epidemiology and Biostatistics, Pontificia Universidad Javeriana, Bogotá, Colombia

**Keywords:** artificial intelligence, cardiovascular risk, decision trees, machine learning, neural networks, random forests, support vector machines

## Abstract

**Introduction:** There is no information on the potential of machine learning (ML)–based techniques to improve cardiovascular risk estimation in the Colombian population. This article presents innovative models using five artificial intelligence techniques: neural networks, decision trees, support vector machines, random forests, and Gaussian Bayesian networks.

**Methods:** The research is based on a cohort of 847 patients free of cardiovascular disease at baseline and followed for cardiovascular disease events over 10 years at the Central Military Hospital in Bogotá, Colombia. To enhance the robustness and reduce the risk of overfitting, model evaluation was conducted using a 5-fold cross-validation on the entire dataset. Discriminatory ability was evaluated with the area under a ROC curve (AUC-ROC) for each ML-based model and the Framingham model.

**Results:** Experimental results showed that the neural network technique had the best discriminative ability to predict cardiovascular events, with an AUC-ROC of 0.69 (CI 95% 0.622–0.759) for unbalanced data and 0.67 (CI 95% 0.601–0.754) for balanced data. Other ML techniques also showed good discriminatory ability with AUC-ROC values between 0.56 and 0.65, superior to that observed for the Framingham model (0.53; CI 95% 0.468–0.607).

**Conclusion:** Our study supports the flexible ML approaches to cardiovascular risk prediction as a way forward for cardiovascular risk assessment in Colombia. Our data even suggest that risk prediction using these techniques could be even more discriminative than widely used risk-stimulation models such as Framingham, adapted to the Colombian population. However, new prospective studies need to validate our data before general implementation.

## 1. Introduction

One of the most significant contributions to cardiovascular epidemiology was the creation of the Framingham study [1] which aimed to detect heart disease at an early stage and to identify subtle manifestations (predisposing factors) in apparently healthy individuals. Since then, various risk stratification models have been proposed to assist physicians in decision-making [2]. These models use risk factors to produce a numerical value (score) that represents the probability of experiencing a cardiovascular event within a given time period. However, according to Cortés et al. [3] and Ridker et al. [4], a significant number of people at risk are not identified by these tools, while others receive unnecessary preventive treatment.

Most vascular risk tables based on quantitative methods are derived from the Framingham study [5]. These scores use a common set of risk factors, namely, age, sex, smoking status, arterial pressure, and lipid levels. Additionally, some scores have integrated more sophisticated markers for cardiovascular disease. However, the addition of new risk factors, while useful in reclassifying those with medium risk above or below a chosen intervention threshold, often has a small effect on the overall model performance measured by the area under the receiver operating characteristic (ROC) curve (AUC-ROC) [6].

Some of the limitations inherent in these models lie in their predominant focus on conventional risk factors, potentially resulting in an underestimation of the influence exerted by emerging factors such as genetics, inflammation, obesity, or low height. Moreover, the constant evolution of knowledge in cardiovascular health may diminish the accuracy of these models in capturing contemporary trends, thereby compromising the reliability of their predictions. Part of this discrepancy may be attributed to the methodological framework underpinning these risk prediction tools, which relies on traditional regression statistics. For instance, the Cox model [1] utilized in the Framingham risk score (with an AUC-ROC of 0.734), the American College of Cardiology/American Heart Association (ACC/AHA) model (with an AUC-ROC of 0.728) [7], the Reynolds risk score (with an AUC-ROC of 0.765) [8], the Prospective Cardiovascular Münster Study (PROCAM) model (with an AUC-ROC of 0.744) [9], or the Weibull model employed in the Systematic Coronary Risk Evaluation (SCORE) model (with an AUC-ROC of 0.63) [10] and adjusted with Fine and Gray competing risk models for SCORE2 [11].

In aiming for the prediction of cardiovascular events, the efficacy of these techniques is constrained by a myriad of underlying assumptions. These include the requirement for linearity in the relationship between independent variables and the logarithm of odds, as well as assumptions of normality, homoscedasticity, and independence within the data. When the problem conforms to these statistical assumptions, the model typically demonstrates robust performance. However, when the interaction between predictor variables and outcomes contravenes these assumptions, the model's ability to generalize predictions to novel cases diminishes significantly [12]. Given these constraints, novel cardiovascular risk models based on machine learning (ML) methodologies have emerged, offering alternative paradigms to traditional logistic or Cox regression models.

Furthermore, these models were developed from specific populations, which may limit their generalizability across different ethnic and geographic cohorts under different health systems. As a result, it is imperative for different countries to either develop their own customized models or conduct calibration studies [13]. In Colombia, several studies have been undertaken to validate:

a. The Framingham and PROCAM models, wherein it was found that despite calibration efforts by the study group, the former exhibited low discriminatory capacity (AUC-ROC of 0.5819), while PROCAM performed more favorably, particularly upon adjustment for sex (AUC-ROC: 0.7446) [14].
b. The ACC/AHA ASCVD score, which demonstrated no significant disparities between expected and observed events and achieved a good discriminatory capacity with an AUC-ROC of 0.782 (95% CI 0.71–0.85) [15].

Moreover, a model known as GLOBORISK-LAC has been developed incorporating a substantial proportion of local Colombian data, which attained a C-statistic of 72%, with calibration slopes of 0.994 for men and 0.852 for women [16]. However, it is crucial to note that this model has not yet undergone validation in other Colombian populations.

In light of the above considerations, there is a striking need to develop a novel strategy to facilitate the construction of a model tailored for predicting cardiovascular risk in the Colombian population. This study advocates the formulation of predictive cardiovascular risk models employing advanced artificial intelligence methodologies, including neural networks, decision trees, support vector machines (SVMs), random forests, and Gaussian Bayesian networks.

## 2. Materials and Methods

### 2.1. Study Design

**Figure 1:**
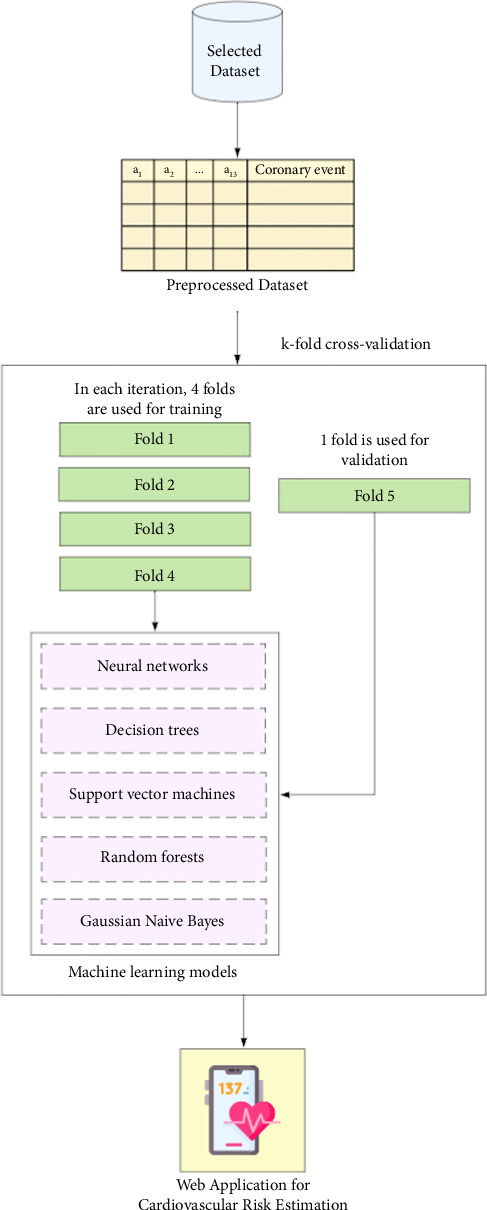
Methodology applied for cardiovascular risk estimation.

Figure 1 illustrates the general methodology employed in this research to derive ML models for cardiovascular risk estimation. The same database used for the validation study of the Framingham and PROCAM models in Colombia was utilized. The population characteristics, operational definitions of the variables, and outcome determinations are fully explained by Muñoz et al. [14]. In brief, the study included patients aged 30–74 years who were free of cardiovascular events at baseline and were followed at the Primary Prevention Clinic of the Central Military Hospital, at Bogotá (Colombia), from 1984 to 2006. Previous studies conducted at the Central Military Hospital have shown that the demographic characteristics and incidence of the most common diseases (including cardiovascular disease) in this population are similar to those reported for the broader Colombian population. This investigation exclusively incorporated clinical variables collected retrospectively, with no specification of names, identification numbers, or other confidential information, thereby obviating the need for informed consent. The authors assert that this research adheres to international standards of biomedical research as per the 64th version of the Helsinki Declaration. The Institutional Research and Ethics Committee of the School of Medicine at the Pontificia Universidad Javeriana approved the study (approval code: FM-CIE-1094-21).

The dataset comprises records from individual patients, each represented by 14 values: 13 independent variables—including age, gender, weight, height, diabetes, systolic and diastolic blood pressure, cholesterol levels, triglycerides, smoking status, and family history of early coronary disease (Table 1)—and one dependent variable. The dependent variable, “Cardiovascular Event,” refers to the diagnosis confirmed by a domain expert physician. To assess model performance and enhance generalizability, a 5-fold cross-validation was applied over the entire dataset. In this procedure, the data are partitioned into five equal subsets; in each iteration, four subsets are used for training and the remaining one for validation. This process is repeated five times so that each subset is used once as the validation set, and the performance metrics are averaged across all folds to provide a more robust estimate of model performance. Five ML techniques were specifically utilized, namely, neural networks, decision trees, SVMs, random forests, and Gaussian Bayesian networks.

Ultimately, the best model was selected based on the AUC-ROC curve. The curve examined the relationship between the true positive rate (TPR) and false positive rate (FPR) by varying classification thresholds. TPR represents the proportion of actual positive cases correctly classified as positive by the model, while FPR indicates the proportion of true negative cases incorrectly classified as positive. A value of 1.0 signifies perfect discriminatory capability, while a value of 0.5 indicates performance similar to randomness. Additionally, we evaluated the mean absolute error (MAE). The MAE calculates the absolute difference between observed values and predicted values, preserving the magnitude of errors without considering their direction. This metric is particularly valuable for evaluating the accuracy of regression models, providing a clear insight into the proximity between predictions and actual values.

Final results were made accessible through a web application, enabling specialist physicians to estimate cardiovascular risk using artificial intelligence. An integral aspect of this article is that the ML models aim to estimate cardiovascular risk by providing an associated risk score. This approach mirrors that of the Framingham score calculation, which similarly considers patient factors in the context of potential cardiovascular events. Finally, the discriminative ability of the ML models was compared with that of traditional statistical methods such as Framingham's.

### 2.2. Dataset and Data Balancing

In this research, a total of 847 records were utilized, with 62 (7.31%) corresponding to patients with positive cardiovascular risk and 785 (92.69%) representing negative diagnoses. This data imbalance primarily stems from patient inclusion based on the presence or absence of cardiovascular risk rather than the occurrence of a cardiovascular event. Unlike other studies that select the population based on whether patients experienced events, such as myocardial infarction, this dataset originates from relatively healthy patients, evaluating the necessity of monitoring them to reduce the incidence of cardiovascular events.

Following attribute selection, an analysis of patient distribution based on the presence of the outcome was conducted. This analysis revealed a significant imbalance between the number of patients who experienced cardiovascular events and those who did not. The utilization of datasets with imbalanced class distribution can introduce biases in ML models. This may cause the model's estimates to lean toward the predominant class in the data, hindering the detection of cases from the minority class. A similar imbalance has been observed in various works addressing the issue of cardiovascular risk prediction [17–20].

Therefore, an oversampling technique called SMOTE-NC [21] was employed to generate additional records. This technique utilizes the difference from its nearest neighbor to insert new values, considering a random number between 0 and 1. Furthermore, it considers both numerical and categorical variables when generating synthetic records. This oversampling technique differs from other approaches as it creates records with subtle variations compared to real data, allowing for a dataset closer to reality and mitigating the impact of class imbalance on the model. To improve data quality and reduce the risk of overfitting due to excessive synthetic data, a partial balancing strategy with a 2:1 ratio was applied. In this configuration, the original 62 instances from the minority class were expanded to 392, while all 785 instances from the majority class were retained, resulting in a balanced dataset of 1177 records, with 330 synthetic instances. This partial oversampling approach maintains a realistic class distribution, helping the model to better detect patterns associated with rare events while preserving the underlying characteristics of the dataset. Compared to complete balancing, the 2:1 ratio offers a compromise that enhances minority class representation without introducing excessive synthetic noise.

### 2.3. ML Models for Cardiovascular Risk Estimation

In this study, Python was employed as the programming language, and scikit-learn [22] served as the ML tool to derive various models for cardiovascular risk estimation. Each technique involves a set of hyperparameters that must be fine-tuned through experimentation to determine a model capable of making predictions with greater accuracy.

#### 2.3.1. Models Obtained With Neural Networks

To obtain models using neural networks, the architecture of the multilayer perceptron for regression outputs (MLP Regressor) was implemented, available in the scikit-learn library of Python [22]. The choice of this implementation was based on the need to obtain continuous values in the range 0–1, as opposed to binary positive or negative responses that might result from other implementations. Manipulating hyperparameters emerges as a crucial aspect to derive various configurations of the neural network intended for cardiovascular risk estimation. In this context, activation functions such as hyperbolic tangent and sigmoid were explored, given the requirement to normalize output values for results within a defined interval. These functions facilitate the generation of bounded values considering the weights and bias of the last hidden layer. Additionally, solvers such as “adam,” “lbfgs,” and “sgd” were employed for the optimization of neural network weights.

**Figure 2:**
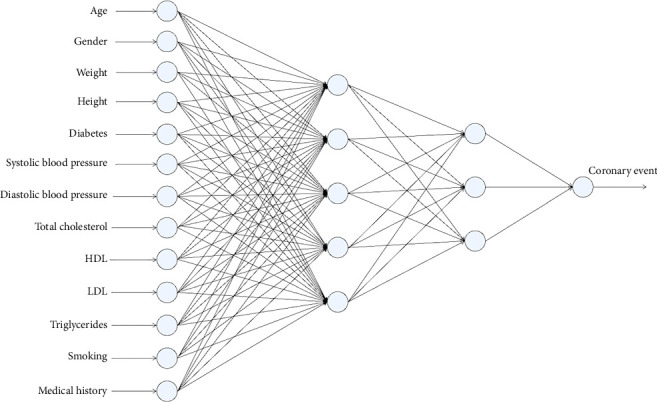
Neural network for cardiovascular risk estimation with topology 13-5-3-1.

To prevent overfitting and improve generalization, L2 regularization was applied through the hyperparameter alpha, which controls the magnitude of the penalty imposed on large weight values. The values explored for alpha were 0.0001, 0.001, 0.01, 0.1, and 1.0, allowing the assessment of different levels of regularization. During the experimental process, networks with 2–5 hidden layers were evaluated, and each layer considered between 1 and 20 nodes. Considering the inclusion of alpha as an additional hyperparameter, a total of 15,000 models were generated.  Figure 2 depicts one of the obtained neural networks with a specific topology of 13-5-3-1. This entails 13 neurons in the input layer corresponding to independent variables, two hidden layers with five and three neurons, respectively, and an output layer with a single neuron.

**Figure 3:**
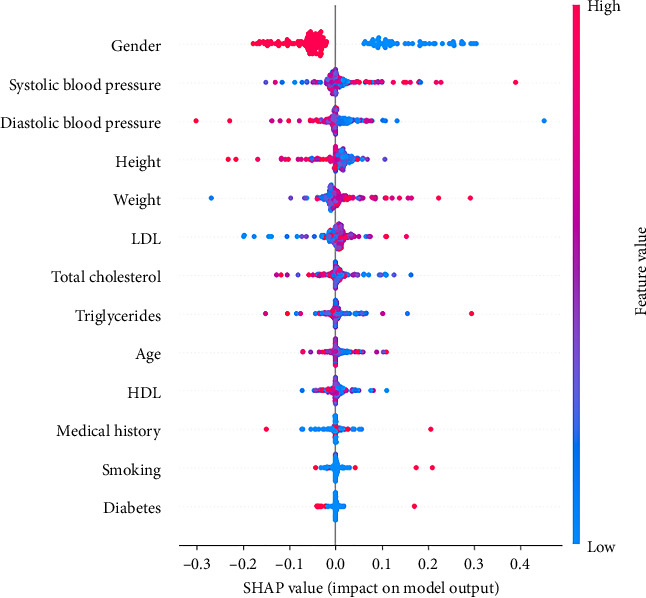
Feature importance analysis using the SHAP technique. The variables are ordered from top to bottom according to their importance in the model's predictions, as measured by the mean absolute SHAP value. This metric represents the average magnitude of a variable's contribution to the prediction, regardless of whether its effect is positive or negative. Higher SHAP values indicate greater influence on the model's decision. In general, SHAP values can be either positive or negative, signifying whether a feature increases or decreases the likelihood of the predicted outcome. The *X*-axis represents the average impact of each variable on the model's output. The points correspond to individual observations from the dataset, illustrating the variability in feature influence across different cases. For instance, gender is the most influential variable in the model's predictions. The data suggest that being male (gender = 0) tends to decrease the likelihood of the predicted condition, whereas being female (gender = 1) significantly increases it, with SHAP values exceeding 0.3 in some cases. Other variables, such as systolic blood pressure, height, and diastolic blood pressure, also have a notable impact, though their influence is lower compared to gender. Conversely, features such as smoking, medical history, and diabetes exhibited relatively minor contributions. The colors in Figure 3 represent the actual values of each feature, providing further interpretability. A blue dot indicates a lower feature value, while a red dot represents a higher value. This allows for a direct visualization of how different ranges of a variable influence the model's prediction. For example, in the case of systolic blood pressure, higher values (red) are generally associated with increased SHAP values, suggesting a positive contribution to the likelihood of the predicted condition. Conversely, lower values (blue) tend to have a negative or negligible impact. This color-coded representation enhances the understanding of how specific feature values drive the model's decisions.

To enhance the interpretability of the model and better understand the factors influencing its predictions, we employed SHapley Additive exPlanations (SHAP). This technique provides insights into how each variable contributes to the model's output, offering a more transparent and explainable decision-making process.  Figure 3 presents a summary of the SHAP values for each feature in the dataset.

#### 2.3.2. Models Obtained With Decision Trees

**Figure 4:**
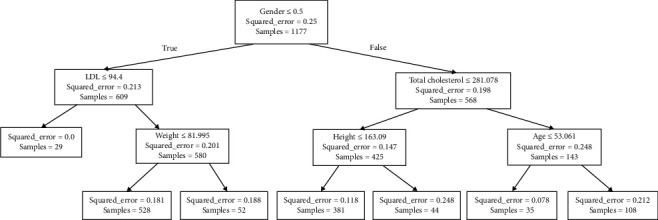
Decision tree for cardiovascular risk estimation.

Decision trees represent a valuable strategy for making estimations in datasets, standing out for their interpretability compared to other techniques. This feature holds particular significance for medical professionals, providing them with the ability to justify scores assigned to each patient. In this context, the scikit-learn library [22] offers the DecisionTreeRegressor implementation of this technique, enabling the generation of regression outputs ranging from 0 to 1 based on the training set. During hyperparameter tuning, different criteria were explored, such as “squared_error,” “Friedman_mse,” “absolute_error,” and “poisson,” to optimize the model's performance. Additionally, the max_depth hyperparameter was varied in a range from 10 to 500 to examine its impact on the tree's predictive ability. Ultimately, strategies for attribute selection at each node were evaluated, considering the options “best” and “random.” This comprehensive exploration led to the assessment of a total of 3000 models.  Figure 4 illustrates a representative example of a decision tree with a depth of three, providing a visual and accessible insight into the structure and decisions made by the model in this specific context. To assign a score to a new patient, the attributes at each node are evaluated, and the branch path is followed based on the decisions made. For instance, if a male patient (encoded as 0) with an LDL level of 96 mg/dL and a weight of 70 kg is considered, the resulting estimation would be 0.763.

#### 2.3.3. Models Obtained With SVMs

The models developed through the application of the SVM technique were obtained using the sklearn.svm.SVR module [22]. Throughout the experimental process, a thorough adjustment of hyperparameters was conducted to fine-tune the model's performance. Specifically, various kernels were explored, including linear, radial basis function, and sigmoid. Adjustments were made to the gamma and penalty coefficient C hyperparameters, utilizing random floating-point values in the range of 0–1. The Coef0 hyperparameter, responsible for controlling the position of the decision boundary in the sigmoid kernel, varied between −100 and 100 by introducing random floating-point values. Finally, the options true and false were explored for the shrinking hyperparameter, indicating whether a shrinking heuristic is employed in SVM optimization. This configuration aims to identify and eliminate elements on the decision boundary, addressing a more manageable optimization problem. The combination of all these hyperparameter modifications led to the evaluation of a total of 10,000 SVM models, seeking the optimal configuration to maximize the system's performance.

#### 2.3.4. Models Obtained With Random Forests

The technique of random forests is grounded in the principle of ensemble learning, a process that combines multiple classifiers to address complex problems and enhance model accuracy. By amalgamating individual models, classification becomes more flexible, characterized by lower bias and less sensitivity to data variations, resulting in reduced variance. In the context of random forests, classification is executed based on predictions from individual decision trees, utilizing the average of the outputs from these trees.

To conduct this research, the RandomForestRegressor classifier from the scikit-learn library was utilized [22]. Among the most critical hyperparameters are the number of trees (n_estimators) used in the ensemble and the criterion parameter, which determines the function to measure the quality of a split. The criterion parameter was varied using four options: “squared_error,” “friedman_mse,” “absolute_error,” and “poisson.” During the experimental phase, the number of trees was adjusted from 10 to 500 in increments of 10, while the max_depth hyperparameter varied from 10 to 200, also in increments of 10. The combination of all these hyperparameter modifications resulted in the evaluation of a total of 4000 models, aiming to identify the optimal configuration that maximizes the performance of the random forest in the context of the research.

#### 2.3.5. Models Obtained With Gaussian Bayesian Networks

Another technique employed to obtain models facilitating cardiovascular risk estimation is Gaussian Bayesian networks, which are grounded in Bayes' theorem and conditional probabilities. The scikit-learn library [22] provides various implementations of this technique, adapted according to the binary or discrete nature of attributes. Throughout this research, exhaustive initial tests were conducted to select the implementation most suitable for the distribution of the training set, resulting in the choice of the standard derivation called Gaussian Naive Bayes (GaussianNB).

The GaussianNB classifier features a single hyperparameter, known as the smoothing variable, enabling the adaptation of various models without the use of randomness. To explore its impact, 3000 instances were generated in which this variable was adjusted, thereby contributing to smoothing the curve and, in some instances, enhancing the classification capability. Although this technique generates binary or classification responses, it is essential to note that the resulting probabilities from each test can be used as numerical outputs, allowing their consideration as an estimation rather than an absolute diagnosis. This approach offers valuable insight by providing continuous information on the probability of belonging to a particular category, thus enriching the interpretation of the obtained results.

## 3. Results

The results are displayed using both the balanced and imbalanced datasets. Furthermore, a comparative analysis is conducted through the AUC-ROC and illustrative graphs comparing the Framingham scale with the scores obtained by the models proposed in this research.

### 3.1. Framingham Risk Score

**Figure 5:**
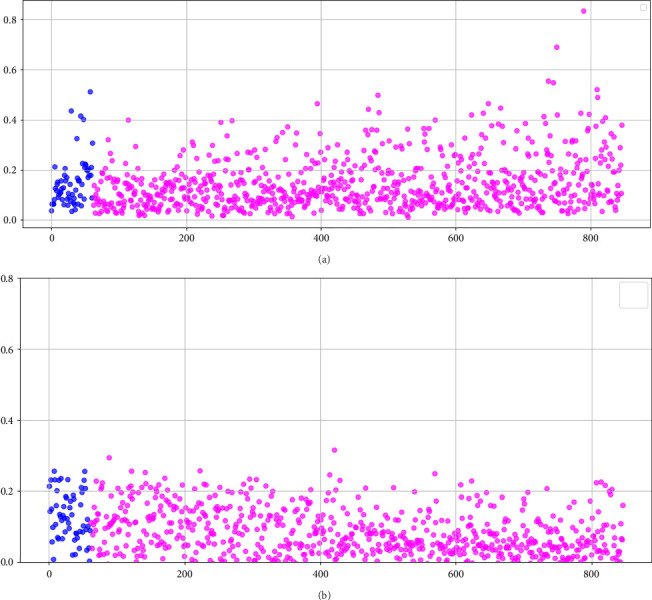
(a) Distribution of risk scores according to the Framingham scale using the unbalanced dataset. The *X*-axis represents the instance number (0–847), and the *Y*-axis corresponds to the risk scores assigned by the Framingham method. Blue points indicate the 62 patients who experienced coronary events, whereas magenta points represent the 785 individuals without such events. (b) Risk predictions obtained from the neural network model applied to the unbalanced dataset. The *X*-axis shows the 847 patient instances, and the *Y*-axis displays the scores predicted by the neural network, ranging from 0 to 1.

Before testing the proposed artificial intelligence models, the Framingham risk score, adjusted for the Colombian population [14], was applied to calculate the AUC-ROC for both the 847 instances in the unbalanced dataset and the 1177 instances in the balanced dataset. For the unbalanced dataset, the AUC was 0.538 (95% CI: 0.468–0.607), while for the balanced dataset, the AUC was 0.519 (95% CI: 0.482–0.551).  Figure 5(a) (unbalanced data) illustrates that the Framingham method assigned high risk scores to individuals who did not experience a cardiovascular event, while individuals who did experience the event were assigned low scores, typically in the range of 0–0.2. The presence of patients who suffered a coronary event but whose risk scores do not accurately reflect their condition highlights the limited discriminatory power of the model.

### 3.2. Neural Networks

Table 2 presents the configurations of neural networks that achieved the top five results based on the AUC-ROC, for both the imbalanced dataset and the dataset balanced using the SMOTE-NC technique. For the imbalanced dataset, the highest AUC of 0.690 (95% CI: 0.622–0.759) was obtained using a tanh activation function, the Adam solver, a network architecture with three hidden layers containing 17, 11, and 18 neurons, and an alpha parameter of 0.1. For the balanced dataset, the highest AUC achieved was 0.677 (95% CI: 0.601–0.754) with a tanh activation function, the SGD solver, a network topology with two hidden layers containing 17 and 10 neurons, and an alpha parameter of 0.01.  Figure 5(b) displays the distribution of estimations from one of the neural networks employed in this study, demonstrating superior performance compared to the Framingham score. However, it is important to note that the model exhibits certain limitations, suggesting the need for further refinement to enhance its accuracy in identifying individuals at risk.

An analysis of the top five neural networks revealed that L2 regularization, controlled by the hyperparameter alpha, played a significant role in the performance of the models. The most accurate network, which reached an AUC-ROC of 0.690 (95% CI: 0.622–0.759), used an alpha value of 0.1. Other high-performing configurations used alpha values of 0.01, 0.001, and even 1.0, suggesting that moderate to strong levels of regularization contributed positively to model generalization. Notably, no top-performing models were associated with the lowest alpha value (0.0001), indicating that minimal regularization may not have been sufficient to prevent overfitting in this context. This behavior is consistent with the complexity of the models and the limited size of the dataset, where an adequate penalization of large weights helps prevent the model from fitting noise in the training data.

### 3.3. Decision Trees

Table 3 presents the selected hyperparameters for the decision trees that achieved the top five results based on the AUC-ROC. For the unbalanced dataset, a decision tree using the Poisson criterion, a maximum depth of 12, and a random splitter achieved an AUC of 0.637 (95% CI: 0.565–0.716), outperforming the Framingham score. Similarly, for the balanced dataset, a decision tree with the Poisson criterion, a maximum depth of 10, and the best splitter achieved an AUC-ROC of 0.656 (95% CI: 0.589–0.721). However, it is important to note that in both the unbalanced and balanced datasets, decision trees exhibited lower AUC values compared to those obtained using neural networks.

The feature importance tool provided by the scikit-learn library was utilized to identify the attributes that play a more significant role in making estimations through this artificial intelligence technique. The most determining variables in the assessment of cardiovascular risk, listed in decreasing order of importance, were age, gender, triglycerides, height, weight, medical history, diastolic blood pressure, LDL, systolic blood pressure, cholesterol, smoking, diabetes, and HDL.

### 3.4. SVMs

Table 3 presents the key hyperparameters associated with the SVM technique that yielded the top five results based on the AUC-ROC. For the imbalanced dataset, the SVM with a sigmoid kernel, a regularization parameter C of 0.67, a gamma coefficient of 0.01, a coef0 of −11.1, and the shrinking hyperparameter set to true, achieved an AUC of 0.648 (95% CI: 0.576–0.718), demonstrating strong performance. In contrast, for the balanced dataset, the configuration of the SVM with a sigmoid kernel, a regularization parameter C of 0.75, a gamma coefficient of 0.01, a coef0 of −11.1, and the shrinking hyperparameter set to false, yielded an AUC of 0.651 (95% CI: 0.577–0.721), indicating its effectiveness in handling balanced data.

### 3.5. Random Forests

Table 3 presents the hyperparameters corresponding to the top five results based on the AUC-ROC when applying the random forest technique to both the imbalanced and balanced datasets. The optimal performance, achieved with the balanced dataset, results in an AUC-ROC of 0.607 (95% CI: 0.533–0.682). This outcome is obtained using 20 trees, a maximum depth of 10, and square error to evaluate the quality of the splits. In contrast, an anomalous behavior was observed when using the imbalanced dataset, where there was a consistent repetition of estimation values, which undermined the reliability of the model for this research. Consequently, this led to the lowest AUC-ROC recorded among all the techniques evaluated, with a value of 0.578 (95% CI: 0.501–0.661), which is comparable to the results obtained using the Framingham method.

### 3.6. Gaussian Bayesian Networks

Table 4 presents the top five results based on the AUC-ROC for the application of Bayesian networks to both the imbalanced and balanced datasets. In the case of the imbalanced dataset, a unique hyperparameter stands out, revealing multiple configurations that achieve the same AUC value of 0.593 (95% CI: 0.522–0.667). For the balanced dataset, an AUC-ROC of 0.579 (95% CI: 0.517–0.648) is obtained using different configurations of the smoothing parameter.

## 4. Discussion

In general, the five ML techniques evaluated in this study for cardiovascular risk assessment exhibited an acceptable ability to discriminate between patients who experienced cardiovascular events and those who did not, surpassing the performance of the widely used Framingham scale. Among these, neural networks stood out, achieving an AUC-ROC of 0.690 (95% CI: 0.622–0.759). This study constitutes a significant contribution in the Colombian context, as it is the first to address the challenge of cardiovascular risk estimation using artificial intelligence techniques. The results are promising and suggest a potential for meaningful impact on clinical decision-making.

The outcomes elucidate the pioneering potential of artificial intelligence in addressing intricate medical challenges such as cardiovascular prediction. Our findings align with a recent meta-analysis conducted by Liu et al. [23], which juxtaposed ML against conventional methodologies for forecasting atherosclerotic cardiovascular risk in primary prevention cohorts. The meta-analysis concluded that ML models exhibit statistically superior discriminative capability, as quantified by Harrell's C statistic, compared to traditional risk assessment tools. This observation remained robust across varying levels of bias risk. However, the assessment of calibration and net reclassification improvement was hindered by the absence of calibration metrics in several studies.

Although the AUC-ROC values obtained in this study are generally lower than those reported in other research [17–19, 24–34], in several cases, they reach comparable levels. For example, Quesada et al. [29] reported an AUC-ROC of 0.708 with Bayesian networks and 0.704 with neural networks in a Spanish population. In our study, the application of neural networks to a Colombian population resulted in an AUC-ROC of 0.690 (95% CI: 0.622–0.759), a value close to that reported in the Spanish context. Likewise, Alaa et al. [17] reported an AUC-ROC of 0.774 using SVMs, random forests, and neural networks on the UK population. Although our values are lower, they reflect the potential of these ML techniques when applied to local data, considering differences in demographic and clinical characteristics across populations.

Finally, as mentioned earlier, there are very few works where any kind of software is developed to enable qualified medical personnel to use the proposed models, limiting the practical application of artificial intelligence models for decision-making in the medical field. To address this limitation, a web application for estimating cardiovascular risk in the Colombian population was proposed. As an integral part of this study, a web application specifically designed for healthcare professionals has been conceptualized and developed. The purpose of this application is to provide healthcare professionals with a simple tool to use the neural network model (that demonstrated the best discrimination capacity) for cardiovascular risk estimation.

**Figure 6:**
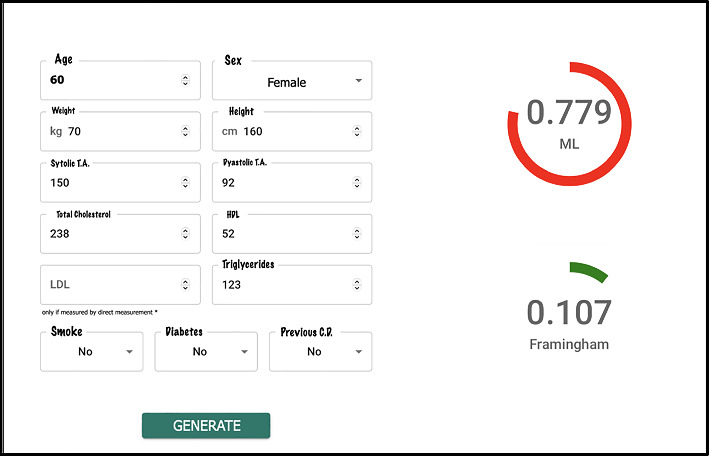
Home page of the cardiovascular risk application.

Figure 6 depicts the home page of the application, where healthcare professionals can input the 13 independent variables used as input to the model, detailed in Table 1. After reading and accepting the privacy policy, the healthcare professional can click the “generate estimation” button. As a result, the score obtained by the neural network is displayed on the right side of the screen. In the visual example shown in Figure 6, this value is 0.799. Additionally, the value obtained according to the Framingham scale is provided, offering the medical professional additional information to support the decision-making process. This comprehensive approach not only facilitates the interpretation of the result provided by the neural network but also enables healthcare professionals to compare and contextualize scores in relation to the reference established by the Framingham scale, thus enhancing the analysis and assessment of the patient's cardiovascular risk.

There are some limitations that need to be recognized. First, our study was developed with data from patients followed up 2 decades ago, so our results need to be externally validated in different settings that represent the actual conditions of the Colombian health system. Beside, a direct comparison with currently used models such as the ACC/AHA ASCVD score [7] and the SCORE2 [11] are needed to confirm our conclusions. Second, the ML techniques used may not behave similarly in new populations, as they tend to overfit the original data, so external validation is also needed in contemporary settings. Finally, our data do not have a significant representation of certain ethnic groups with high prevalence in Colombia, for example Afro-Colombian or indigenous populations represent 14% of the Colombian population but are not represented in our study, so our data cannot be used in these populations without prior verification of our conclusions. Indeed, there is still work to be performed to ensure that they are suitable for implementation, including new studies developed in prospective cohort studies, nonrandomized controlled trials, among others.

## 5. Conclusions

In conclusion, our study supports the notion that flexible ML approaches for cardiovascular risk prediction could be the way for enhanced cardiovascular risk assessment in Colombia, taking advantage of an increasingly data-rich world. Our data even suggest that risk prediction using these techniques could be even more discriminative than widely used risk-stimulation models such as Framingham's, adapted to the Colombian population. However, new prospective studies need to validate our data before generalized implementation.

## Figures and Tables

**Table 1:** Attributes used for cardiovascular risk estimation.

Index	Variable	Definition	Variable type	Operational level
1	Age	Time between the date of birth and the date of entry into the record	Discrete quantitative	(30–74)
2	Gender	Patient's gender	Nominal qualitative	0 = male 1 = female
3	Weight	Patient's weight	Continuous quantitative	(41–102)
4	Height	Patient's height	Continuous quantitative	(136–197)
5	Diabetes	Indicates whether the patient suffers from diabetes	Nominal qualitative	0 = negative 1 = positive
6	Systolic blood pressure	Measurement of systolic blood pressure	Continuous quantitative	(90–230)
7	Diastolic blood pressure	Measurement of diastolic blood pressure	Continuous quantitative	(60–140)
8	Total cholesterol	Measurement of the total amount of cholesterol in the blood	Continuous quantitative	(98–478)
9	HDL	Measurement of the amount of HDL cholesterol in the blood	Continuous quantitative	(18.1–100)
10	LDL	Measurement of the amount of LDL cholesterol in the blood	Continuous quantitative	(5.8–386.4)
11	Triglycerides	Measurement of the amount of triglycerides in the blood	Continuous quantitative	(40–932)
12	Smoking	Tobacco or similar substance consumption	Nominal qualitative	0 = negative 1 = positive
13	Family history	Indicates whether first-degree relatives have experienced any coronary event before the age of 60	Nominal qualitative	0 = negative 1 = positive
14	Coronary event	Confirmed coronary heart disease: Cardiovascular death, acute myocardial infarction, angina pectoris, or coronary insufficiency	Nominal qualitative	0 = negative 1 = positive

**Table 2:** Results obtained by the top five estimation models using neural networks.

Activation function	Solver	Topology of hidden layers	Alpha	MAE	AUC-ROC (95% CI)
*Unbalanced dataset*					
Tanh	Adam	17-11-18	0.1	0.138	0.690 (0.622–0.759)
Logistic	Lbfgs	3-8	0.1	0.141	0.666 (0.598–0.730)
Tanh	Lbfgs	20-19-16-4	0.1	0.134	0.665 (0.597–0.727)
Logistic	Adam	17-15-2-4	0.001	0.130	0.663 (0.593–0.730)
Logistic	Adam	12-20-8-8	0.01	0.137	0.662 (0.596–0.728)

*Balanced dataset*					
Tanh	sgd	17-10	0.01	0.271	0.677 (0.601–0.754)
Tanh	Adam	9-12	1.0	0.248	0.675 (0.606–0.742)
Tanh	Adam	11-1	0.0001	0.251	0.671 (0.605–0.735)
Tanh	Adam	2-4	0.001	0.272	0.668 (0.602–0.733)
Tanh	sgd	19-7-16	0.1	0.263	0.661 (0.595–0.718)

**Table 3:** Results obtained by the top five estimation models using different ML models.

ML models	Criterion	Maximum depth	Splitter	MAE		AUC-ROC (95% CI)	
Decision trees	*Unbalanced dataset*						
	Poisson	12	Random	0.133		0.637 (0.565–0.716)	
	Poisson	11	Best	0.126		0.627 (0.562–0.687)	
	Poisson	11	Random	0.142		0.627 (0.556–0.692)	
	Poisson	10	Best	0.127		0.624 (0.559–0.684)	
	Poisson	13	Random	0.137		0.620 (0.554–0.690)	
	*Balanced dataset*						
	Poisson	10	Best	0.192		0.656 (0.589–0.721)	
	Poisson	13	Best	0.181		0.649 (0.583–0.714)	
	Poisson	16	Best	0.176		0.648 (0.581–0.713)	
	Poisson	17	Best	0.175		0.648 (0.581–0.713)	
	Poisson	14	Best	0.181		0.648 (0.581–0.710)	

	**Kernel**	**C**	**Gamma**	**Coef 0**	**Shrinking**	**MAE**	**AUC-ROC (95% CI)**

Support vector machines	*Unbalanced dataset*						
	Sigmoid	0.67	0.01	−11.1	True	0.159	0.648 (0.576–0.718)
	Sigmoid	0.67	0.01	−11.1	False	0.159	0.648 (0.576–0.718)
	Sigmoid	0.51	0.01	−11.1	True	0.159	0.648 (0.576–0.718)
	Sigmoid	0.59	0.01	−11.1	False	0.159	0.648 (0.576–0.718)
	Sigmoid	0.59	0.01	−11.1	False	0.159	0.648 (0.576–0.718)
	*Balanced dataset*						
	Sigmoid	0.75	0.01	−11.1	False	0.159	0.651 (0.577–0.721)
	Sigmoid	0.75	0.01	−11.1	True	0.159	0.651 (0.577–0.721)
	Sigmoid	0.34	0.01	−11.1	True	0.159	0.651 (0.577–0.721)
	Sigmoid	0.34	0.01	−11.1	False	0.159	0.651 (0.577–0.721)
	Sigmoid	0.42	0.01	−11.1	False	0.159	0.651 (0.577–0.721)

	**Criterion**		**Maximum depth**		**Number of trees**	**MAE**	**AUC-ROC (95% CI)**

Random forests	*Unbalanced dataset*						
	Friedman_mse		10		10	0.145	0.578 (0.501–0.661)
	Squared_error		10		10	0.145	0.578 (0.501–0.661)
	Friedman_mse		10		30	0.143	0.572 (0.488–0.656)
	Squared_error		10		30	0.143	0.572 (0.488–0.656)
	Squared_error		10		220	0.145	0.568 (0.482–0.654)
	*Balanced dataset*						
	Squared_error		10		20	0.199	0.607 (0.533–0.682)
	Friedman_mse		10		20	0.199	0.607 (0.533–0.682)
	Squared_error		10		50	0.203	0.602 (0.527–0.674)
	Friedman_mse		10		50	0.203	0.602 (0.527–0.674)
	Absolute error		173		130	0.224	0.601 (0.523–0.677)

**Table 4:** Results obtained by the top five estimation models using Bayesian networks.

Smoothing variable	MAE	AUC-ROC (95% CI)
*Unbalanced dataset*		
1.00e − 07	0.105	0.593 (0.522–0.667)
1.00e − 12	0.105	0.593 (0.522–0.667)
1.02e − 12	0.105	0.593 (0.522–0.667)
1.01e − 12	0.105	0.593 (0.522–0.667)
1.03e − 12	0.105	0.593 (0.522–0.667)

*Balanced dataset*		
1.00e − 07	0.312	0.579 (0.517–0.648)
1.00e − 12	0.312	0.579 (0.517–0.648)
1.01e − 11	0.312	0.579 (0.517–0.648)
1.01e − 12	0.312	0.579 (0.517–0.648)
1.02e − 12	0.312	0.579 (0.517–0.648)

## Data Availability

The data that support the findings of this study are available from the corresponding author upon reasonable request.
